# Cost Shifting for Emergency Care of Veterans With Medicare After MISSION Act Implementation

**DOI:** 10.1001/jamahealthforum.2024.4312

**Published:** 2024-12-27

**Authors:** Laura G. Burke, Yanlei Ma, Jessica Phelan, Ellen Latsko, Austin B. Frakt, Steven D. Pizer, Jose F. Figueroa

**Affiliations:** 1 Department of Emergency Medicine, Beth Israel Deaconess Medical Center, Harvard Medical School, Boston, Massachusetts; 2 Department of Health Policy and Management, Harvard T.H. Chan School of Public Health, Boston, Massachusetts; 3 Partnered Evidence-based Policy Resource Center, Boston Veterans Affairs Healthcare System, Boston, Massachusetts; 4 Department of Health Law, Policy and Management, Boston University School of Public Health, Boston, Massachusetts

## Abstract

This cross-sectional study examines changes in payers for emergency department visits in Veterans Affairs and community facilities from 2016 to 2021.

## Introduction

In 2018, US Congress enacted the Maintaining Internal Systems and Strengthening Integrated Outside Networks (MISSION) Act, broadening veterans’ access to community care.^1,2^ Since its 2019 implementation, there has been a marked increase in community emergency department (ED) visits paid by the US Department of Veterans Affairs (VA) and in VA spending on community ED care.^3^ An important dynamic arises among veterans eligible for VA coverage (eg, due to service-connected disability) and also enrolled in Medicare. Given that community care can be paid by the VA or Medicare, there is potential for payer substitution or cost shifting for ED care from Medicare to the VA.^4^ Understanding changes in ED payer source post–MISSION Act implementation is critical to inform national efforts to improve health care delivery for veterans and ensure optimal use of federal funds.

## Methods

Using linked Medicare-VA data between January 1, 2016, and December 31, 2021, we identified veterans (aged ≥18 years) covered by both programs. In accordance with the Common Rule, this cross-sectional study was exempt from ethics review and informed consent requirement because it was classified as nonresearch by the VA. We followed the STROBE reporting guideline.

From the Program Integrity Tools database, we obtained the number of VA ED visits at VA or community facilities purchased by the VA.^5^ Using ED revenue center codes from traditional Medicare (TM) and Medicare Advantage (MA) data, we identified Medicare-purchased community ED visits. We calculated the number of yearly ED visits per 1000 veterans in Medicare overall and by VA ED visits, VA-purchased community ED visits, and Medicare-purchased community ED visits. We ascertained yearly percentage of ED visits across these 3 categories. We estimated total costs shifted from Medicare to VA post–MISSION Act implementation (eMethods in Supplement 1). Data analysis was performed from February 1, 2023, to September 18, 2024, with SAS, version 9.4 (SAS Institute).

## Results

**Figure 1.**
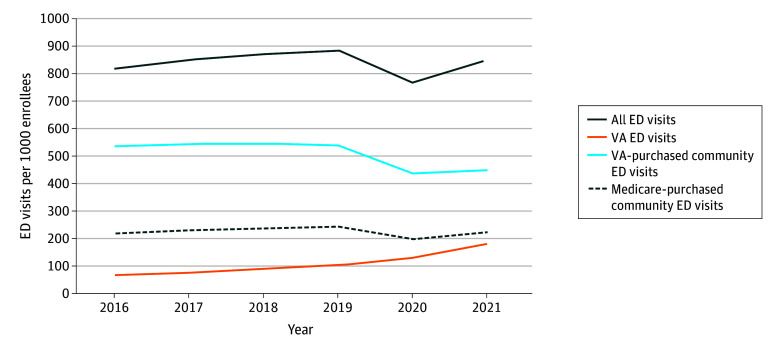
Emergency Department (ED) Visits, by Payer and Site of Care, per 1000 Veterans Dually Enrolled in Medicare and the US Department of Veterans Affairs (VA) All individuals were enrolled in the VA and either traditional Medicare or Medicare Advantage. Inpatient and outpatient ED visits were identified using VA data as well as traditional Medicare and Medicare Advantage claims.

There were 4 960 189 VA and Medicare enrollees in 2016 and 4 837 436 in 2021 (4 624 157 males [95.6%], 213 279 females [4.4%]; mean [SD] age, 74.1 [9.9] years). The percentage with at least 1 ED visit was 37.0% in 2016 and 37.6% in 2021. ED visits increased 8.0% from 820 per 1000 veterans in 2016 to 886 per 1000 veterans in 2019 (Figure 1). In 2020, during the COVID-19 pandemic, ED visits decreased to 769 per 1000 veterans but increased to 852 per 1000 veterans in 2021.

**Figure 2.**
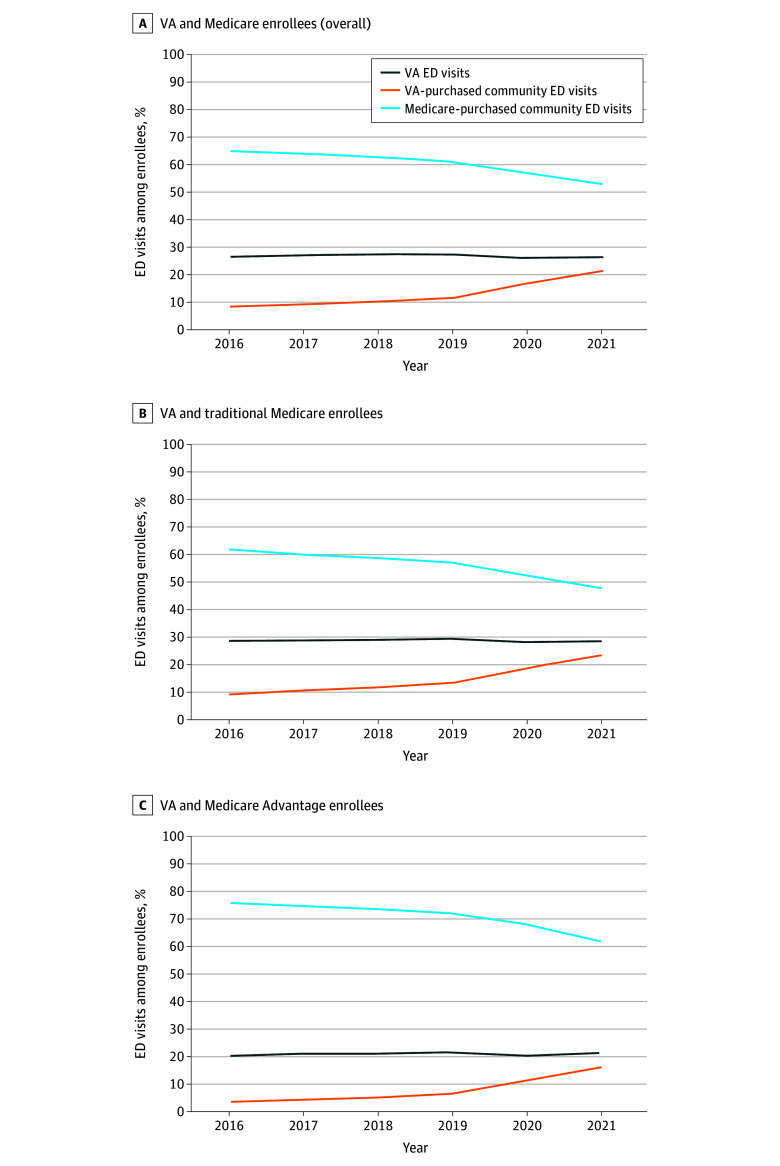
Proportion of Emergency Department (ED) Visits Paid by the US Department of Veterans Affairs (VA) and by Medicare, Overall and Stratified by Traditional Medicare vs Medicare Advantage From 2016 to 2021 All individuals were enrolled in the VA and either traditional Medicare or Medicare Advantage. Inpatient and outpatient ED visits were identified using VA data as well as traditional Medicare and Medicare Advantage claims.

When stratifying by site and payer, VA-purchased community ED visits increased 172.7% from 66 per 1000 veterans in 2016 to 180 per 1000 veterans in 2021. Medicare-purchased community ED visits were increasing pre–MISSION Act implementation but decreased 17.9% from 546 per 1000 veterans in 2018 to 448 per 1000 veterans in 2021. The percentage of all VA ED visits remained stable from 26.8% in 2016 to 26.3% in 2021 (Figure 2). Between 2016 and 2021, the percentage of VA-purchased community ED visits increased from 8.0% to 21.1%, while Medicare-purchased community ED visits decreased from 65.2% to 52.6%. Patterns were similar among veterans enrolled in TM and MA. We estimated that at least $2 billion of VA community ED spending in 2021 was due to payer shift from Medicare.

## Discussion

Between 2016 and 2021, there was a marked decrease in Medicare-purchased community ED visits and a concomitant increase in VA-purchased community ED visits. Results suggest a substitution effect and cost shifting from Medicare to VA following MISSION Act implementation, which we estimated as approximately $2 billion in 2021. This shift is particularly concerning among veterans enrolled in MA since plans receive capitated payments regardless of actual use of VA- or Medicare-covered services.^6^ Policymakers and VA leaders should consider health reforms to improve care efficiency for veterans.

This study’s observational design limited our ability to infer causality between MISSION Act implementation and payer change. Nevertheless, using comprehensive data on Medicare and VA enrollees, this study showed that costs of community ED care for veterans are shifting from Medicare to the VA.

## Notes

Supplement 1. **eMethods.** Methods for Estimating Cost Shifting From Medicare to the Veterans Health Administration (VA) After the MISSON Act of 2018

Supplement 2.
Data Sharing Statement

## References

[1] Massarweh NN, Itani KMF, Morris MS. The VA MISSION Act and the future of veterans’ access to quality health care. JAMA. 2020;324(4):343-344. doi:10.1001/jama.2020.4505 32602896

[2] Getting emergency care at non-VA facilities. US Dept of Veterans Affairs website. Accessed March 6, 2024. https://www.va.gov/resources/getting-emergency-care-at-non-va-facilities/

[3] Vashi AA, Urech T, Wu S, Tran LD. Community emergency care use by veterans in an era of expanding choice. JAMA Netw Open. 2024;7(3):e241626. doi:10.1001/jamanetworkopen.2024.1626 38457180 PMC10924239

[4] Rose L, Tran D, Wu S, Dalton A, Kirsh S, Vashi A. Payer shifting after expansions in access to private care among veterans. Health Serv Res. 2023;58(6):1189-1197. doi:10.1111/1475-6773.14162 37076113 PMC10622298

[5] Community care data: Program Integrity Tool (PIT). US Dept of Veterans Affairs website. Accessed September 7, 2024. https://www.herc.research.va.gov/include/page.asp?id=choice-pit

[6] Trivedi AN, Grebla RC, Jiang L, Yoon J, Mor V, Kizer KW. Duplicate federal payments for dual enrollees in Medicare Advantage plans and the Veterans Affairs health care system. JAMA. 2012;308(1):67-72. doi:10.1001/jama.2012.7115 22735360 PMC4772733

