# Errors in Figure

**DOI:** 10.1001/jamahealthforum.2024.5633

**Published:** 2025-02-07

In the Research Letter titled “Cost Shifting for Emergency Care of Veterans With Medicare After MISSION Act Implementation,”^1^ which published December 27, 2024, the labels in the key for Figure 1 were incorrectly placed. This article has been corrected.
