# Supplementary material for: Cost Shifting for Emergency Care of Veterans With Medicare After MISSION Act Implementation
Source: JAMA Health Forum. 2024 Dec 27;5(12):e244312. doi: 10.1001/jamahealthforum.2024.4312 (PMC11681371; doi:10.1001/jamahealthforum.2024.4312)
Supplement: Supplement 1. — eMethods. Methods for Estimating Cost Shifting From Medicare to the Veterans Health Administration (VA) After the MISSON Act of 2018 [file jamahealthforum-e244312-s001.pdf]

Supplemental Online Content

Burke LG, Ma Y, Phelan J, et al. Cost shifting for emergency care of veterans with Medicare after MISSION Act implementation. *JAMA Health Forum.* Published online December 27, 2024. doi:10.1001/jamahealthforum.2024.4312

eMethods. Methods for Estimating Cost Shifting From Medicare to the Veterans Health Administration (VA) After the MISSON Act of 2018

This supplemental material has been provided by the authors to give readers additional information about their work.

© 2024 Burke LG et al. *JAMA Health Forum*.

eMethods. Methods for estimating cost shifting from Medicare to the Veterans Health Administration (VA) after the MISSON Act of 2018

I. <u>Calculation of Mean Cost of a Community ED Visit</u>

Using the community care claims data from the Consolidated Data Set (CDS) of the VA Office of Integrated Veteran Care in 2021, the average spending per community care ED visit (including associated inpatient hospital stays for admitted patients) was **$4,437**. The method of attributing emergency inpatient stays to the cost of the ED visit is consistent with prior work (Vashi et al;. JAMA Netw Open. 2024;7(3):e241626.)

II. <u>Calculation of the number of visits shifted from Medicare to the VA as the payer</u>

We identified all community ED visits among dual VA/Medicare enrollees in 2018 (the year before the MISSION Act was implemented) as well as in 2021 (the most recent year in our dataset post-MISSION Act). We calculated the percentage of community ED visits attributed to each payer. Of note, visits among enrollees in Traditional Medicare (TM) and Medicare Advantage (MA) were aggregated into one "Medicare" group.

We then applied the 2018 payer distribution to the number of community ED visits in 2021 to calculate the expected number of ED visits by payer in 2021 if payer distribution had not changed after the MISSION Act.

III. <u>Calculation total spending that shifted from Medicare to the VA in 2021</u>

We then multiplied 447,616 ED visits that were calculated as being shifted from Medicare to the VA by the average $4,437 average spending per community ED visit calculated in Step I.

→ We estimate that at least **$2.0 billion** dollar spent by the VA in 2021 on emergency care provided in the community would have been paid by Medicare (traditional Medicare or Medicare Advantage) if payer distribution had remained the same as 2018 (i.e., before the MISSION Act).

© 2024 Burke LG et al. *JAMA Health Forum*.
