# Supplementary material for: Cost Shifting for Emergency Care of Veterans With Medicare After MISSION Act Implementation
Source: JAMA Health Forum. 2024 Dec 27;5(12):e244312. doi: 10.1001/jamahealthforum.2024.4312 (PMC11681371; doi:10.1001/jamahealthforum.2024.4312)
Supplement: Supplement 2. — Data Sharing Statement [file jamahealthforum-e244312-s002.pdf]

Data Sharing Statement

Burke. Cost Shifting for Emergency Care of Veterans With Medicare After MISSION Act Implementation. *JAMA Health Forum*. Published December 27, 2024. doi:10.1001/jamahealthforum.2024.4312

Data
Data available: No

Additional Information
Explanation for why data not available: This study was conducted using VA/Medicare data that is governed by Data Use Agreements that prohibit sharing of the data.
